# Composites Based on Poly(Lactic Acid) (PLA) and SBA-15: Effect of Mesoporous Silica on Thermal Stability and on Isothermal Crystallization from Either Glass or Molten State

**DOI:** 10.3390/polym12112743

**Published:** 2020-11-19

**Authors:** Tamara M. Díez-Rodríguez, Enrique Blázquez-Blázquez, Ernesto Pérez, María L. Cerrada

**Affiliations:** Instituto de Ciencia y Tecnología de Polímeros (ICTP-CSIC), Juan de la Cierva 3, 28006 Madrid, Spain; t.diez@ictp.csic.es (T.M.D.-R.); enrique.blazquez@ictp.csic.es (E.B.-B.); ernestop@ictp.csic.es (E.P.)

**Keywords:** PLA and SBA-15 composites, thermal stability, isothermal crystallization, polymorphism, microhardness

## Abstract

Several composites based on an *L*-rich poly(lactic acid) (PLA) with different contents of mesoporous Santa Barbara Amorphous (SBA-15) silica were prepared in order to evaluate the effect of the mesoporous silica on the resultant PLA materials by examining morphological aspects, changes in PLA phases and their transitions, and, primarily, the influence on some final properties. Melt extrusion was chosen for the obtainment of the composites, followed by quenching from the melt to prepare films. Completely amorphous samples were then attained, as deduced from X-ray diffraction and differential scanning calorimetry (DSC) analyses. Thermogravimetric analysis (TGA) results demonstrated that the presence of SBA-15 particles in the PLA matrix did not exert any significant influence on the thermal decomposition of these composites. An important nucleation effect of the silica was found in PLA, especially under isothermal crystallization either from the melt or from its glassy state. As expected, isothermal crystallization from the glass was considerably faster than from the molten state, and these high differences were also responsible for a more considerable nucleating role of SBA-15 when crystallizing from the melt. It is remarkable that the PLA under analysis showed very close temperatures for cold crystallization and its subsequent melting. Moreover, the type of developed polymorphs did not accomplish the common rules previously described in the literature. Thus, all the isothermal experiments led to exclusive formation of the α modification, and the observation of the α’ crystals required the annealing for long times at temperatures below 80 °C, as ascertained by both DSC and X-ray diffraction experiments. Finally, microhardness (MH) measurements indicated a competition between the PLA physical aging and the silica reinforcement effect in the as-processed amorphous films. Physical aging in the neat PLA was much more important than in the PLA matrix that constituted the composites. Accordingly, the MH trend with SBA-15 content was strongly dependent on aging times.

## 1. Introduction

Poly(lactic acid) (PLA) possesses several attractive characteristics. Among others, it is obtained from renewable resources, and it is biodegradable, biocompatible, and semicrystalline. All of these aspects have led to its use in both specific and commodity applications [1,2].

The monomer of this aliphatic polyester, the lactic acid, presents two stereoisomers *L*- and *D*-lactic acid and, consequently, two dimers named *L*- and *D*-lactide. Commercial PLA grades with the most industrial relevance are those containing only around 1–10% of *D*-lactide. Regardless of the final application, the *D*-lactide content in an *L*-rich PLA (commonly labeled simply as PLA) is essential because it controls characteristics as important as the crystallization rate, transparency, and melting temperature. Its significance becomes even greater once one considers that the overall nucleation and crystallization rates of PLA are relatively low.

Homopolymers of *L*-rich PLA exhibit different crystalline structures: α’, α, β, and γ [3,4,5,6,7]. The α’ and α crystals are developed under most processing conditions depending on thermal history. The β polymorph is generated at elevated temperatures through the deformation of α crystallites [5,6,7]. The γ phase shows a very different chain conformation and packing compared with those found in the α polymorph [4]. In addition, a mesomorphic phase can be obtained by stretching at temperatures lower than around 80 °C [8,9].

The two former α’ and α crystal forms are, however, the most common polymorphs, showing a remarkable similarity. Even so, those subtle structural variations of the α’ and the α phases can lead to dramatic changes in properties. Furthermore, a transformation from the α’ to the α form has also been observed and characterized [10].

In spite of the aforementioned similitude, the crystalline differences between the α’ and the α lattices are related to the fact that the former is consistent with a hexagonal packing by X-ray diffraction (i.e., the ratio of *a*-axis to *b*-axis is $\sqrt{3}$ [11]), while the α crystal displays an orthorhombic unit cell. Moreover, vibrational spectroscopy has shown changes in the conformation and packing of PLA chains for the two crystalline phases [10,11,12,13,14,15,16]. The α phase consists of a 10_3_ helix conformation, while the α’ chains exhibits a distorted helix one [17]. There are also important variations in their heat of fusion [18], although the structural parameters of the two crystalline phases are nearly the same. All of these features suggest that inter-chain interactions, from carbonyl and methyl functional groups, can be quite different. These interactions have been attributed to the relative orientation of the molecular dipoles in the unit cell [15,18].

As discussed, PLA is characterized by relatively low overall nucleation and crystallization rates. Accordingly, numerous investigations have been focused on the enhancement of PLA crystallization kinetics [19] by adding nucleants to increase nucleation density or by incorporating plasticizers to boost chain mobility. Common nucleating agents used up to now include [16,19,20,21,22,23,24,25,26,27]: CaCO_3_, TiO_2_, talc, calcium lactate, carbon nanotubes, graphene oxide, nanoclay, and hydrazides.

Ordered mesoporous silicas can be a suitable alternative to nucleating agents since they can interact with polymeric chains, either from the exterior of the particles or the interior of the empty pores existing in their structures if macromolecules are able to be included in that nanometric space. These silicas are characterized by the presence of well-organized arrangements at long range. Mobil Composition of Matter MCM-41 and Santa Barbara Amorphous SBA-15 are the best-known members, and both are constituted by hollow channels ordered in hexagonal frames [28,29]. Their pore size is the main difference between them, the diameter ranging from 2 to 4 nm in the former [28] and varying from 5 to 10 nm in the latter [29]. These ordered porous silicas on the nanoscale have found diverse applications in several fields, including catalysis, coatings, optics, drug delivery, diagnostics, gas-separation, bio-separation, cosmetics, and nanotechnology.

Composites achieved from the incorporation of different contents of pristine SBA-15 silica into an *L*-rich PLA can then turn out very interesting due to the increasing importance of materials based on PLA and the extensive features of mesoporous silicas. Furthermore, these materials have been hardly evaluated [30]. Interest is even greater if the selected processing technique is melt extrusion since it is a cost-effective and an environmentally-friendly transformation approach that does not require the use of solvents. Therefore, the purpose of this research was to prepare the as-processed amorphous films for materials based on PLA and neat SBA-15 particles by extrusion, as well as to evaluate their thermal stability, crystallization capability (under dynamic or isothermal conditions), and mechanical response. For that, the resultant composites after extrusion were analyzed, with attention paid to the exhibited morphological aspects, the changes in PLA phases and their transitions, and, primarily, the influence of SBA-15 silica on the materials’ final properties (thermal stability or mechanical behavior). Numerous techniques were required in this research, including Transmission electron microscopy (TEM), scanning electron microscopy (SEM), X-ray experiments with conventional and synchrotron radiation, Raman spectroscopy, thermogravimetric analysis (TGA), differential scanning calorimetry (DSC), and microhardness tests. These composites could find applicability by themselves, but their application fields could be even enlarged if these mesostructures were modified in a previous stage. Thus, functionalized composites based on PLA and decorated mesoporous silica could be attained with specific functionalities, such as antimicrobial, drug carrier, and CO_2_ capture features, thus allowing for the achievement of very versatile PLA materials.

## 2. Materials and Methods

### 2.1. Materials and Chemicals

A commercially available PLA from NatureWorks^®^ (Minnetonka, MN, USA) was used in this study. Its grade product was labeled as 3051D, with content in *L*-isomer units of about 96 mol%, a melt flow index of 6.5 g/10 min (190 °C and 2.16 kg), and a density of 1.25 g/cm^3^. Its weight-average molecular weight (*M_w_*) and molecular weight distribution (*M_w_/M_n_*) were 148.6 kg/mol and 1.94, respectively, as determined from gel permeation chromatography (GPC) [9].

The SBA-15 particles were purchased from Sigma-Aldrich (San Luis, MO, USA) (specific surface area, *S*_BET_ = 517 m^2^/g; total pore volume, *V_t_* = 0.83 cm^3^/g; and average mesopore diameter, *D_p_* = 6.25 nm [31]) and were used as received.

### 2.2. Composite and Film Preparation

Composites with different contents of SBA-15 particles (1, 3, 6, and 9% in weight, labeled as PLA-SBA1, PLA-SBA3, PLA-SBA6, and PLA-SBA9, respectively) were processed by melt extrusion in a corotating twin-screw micro extruder (Rondol, Rondol Industrie, Nancy, France) at a rate of 60 rpm. Prior to extrusion, both the polymer and SBA-15 were dried. The former was placed in an oven at 100 °C for 20 min followed by drying under vacuum at 85 °C for 2 h. The SBA-15 particles were dried under vacuum at 100 °C for 24 h. In the extruder, screw temperature profiles of 125, 160, 190, 190 and 185 °C were used from the hopper to the die, with the length-to-diameter ratio being 20:1. Then, films were obtained by compression molding in a hot-plate Collin press (Collin GmbH, Maitenbeth, Germany). The first stage was the maintenance of the material at a temperature of 185 °C and at a pressure of 30 bar for 6 min. After that, a cooling process was applied to the different composites from their molten state to room temperature for 4 min at the relatively fast rate of around 80 °C/min and at a pressure of 30 bar.

### 2.3. Transmission Electron Microscopy

Morphological details of the mesoporous SBA-15 silica were obtained by TEM. Measurements were performed at room temperature with a 200 kV JEM-2100 JEOL microscope (JEOL Ltd., Tokyo, Japan). The particles were dispersed in acetone in an ultrasonic bath for 5 min and then deposited into a holder prior to observation.

### 2.4. Scanning Electron Microscopy

SBA-15 particle dispersion within the PLA matrix was evaluated by high resolution field emission scanning electron microscopy (FESEM). Experiments were carried out with a Hitachi S-8000 (Hitachi Co., Tokyo, Japan) at room temperature in different cryo-fractured sections of composites with distinct mesoporous contents. Those thin sections of around 40 nm were cut by cryo-ultramicrotomy (Leica EM UC6, Leica Microsystems GmbH, Wetzlar, Germany) at −120 °C and deposited in a holder.

### 2.5. X-ray Experiments with Conventional and Synchrotron Radiation

Wide angle X-ray diffraction (WAXD) patterns at room temperature were recorded to examine the crystalline structure of the polymeric matrix in the reflection mode by using a Bruker D8 Advance diffractometer provided with a position-sensitive detector (PSD) Vantec detector (from Bruker, Madison, WI, USA). Cu Kα radiation (*λ* = 0.15418 nm) was used, operating at 40 kV and 40 mA. The parallel beam optics were adjusted by a parabolic Göbel mirror with a horizontal grazing incidence Soller slit of 0.12° and an LiF monochromator. The equipment was calibrated with different standards. A step scanning mode was employed for the detector. The diffraction scans were collected with a 2θ step of 0.024° and 0.2 s per step.

Real-time variable-temperature simultaneous small-angle X-ray scattering (SAXS) and WAXD experiments were carried out with synchrotron radiation in Non Crystalline Diffraction beamline (BL11-NCD) at ALBA (Cerdanyola del Valles, Barcelona, Spain) at a fixed wavelength of 0.1 nm. A Pilatus 1M detector (Dectris Ltd., Baden-Daettwil, Switzerland) was used for SAXS (off beam, at a distance of 296 cm from sample) and a Rayonix one (Rayonix, Evanston, IL, USA) for WAXD (at about 14.6 cm from the sample and a tilt angle of around 29 degrees). A Linkam unit, connected to a cooling system of liquid nitrogen, was employed for the temperature control. The calibration of spacings was obtained by means of silver behenate and Cr_2_O_3_ standards. The initial 2D X-ray images were converted into 1D diffractograms as a function of the inverse scattering vector, *s* = 1/*d* = 2 sin θ/λ, by means of the fast azimuthal integration python (pyFAI) code (European Synchrotron Radiation Facility, ESRF), modified by the ALBA beamline staff. Film samples of around 5 × 5 × 0.1 mm were used in the synchrotron analysis.

### 2.6. Raman Spectroscopy

Knowledge one the conformations and packing of PLA macrochains was gained via Raman spectroscopy. These experiments were performed in the Raman Micro-Spectroscopy Laboratory of the Characterization Service at ICTP-CSIC by using a Renishaw InVia Reflex Raman system (Renishaw plc, Wotton-under-Edge, UK), provided with a grating spectrometer coupled to a confocal microscope, as well as a Peltier-cooled charge-coupled device (CCD) detector. A diode laser (wavelength of 785 nm) was used for exciting the Raman scattering. The laser beam, with a power of 160 mW, was focused at the sample with the aid of a 0.85 × 100 microscope objective. An exposure time of 1 s and 200 accumulations were employed in these Raman measurements. After a baseline correction, the Raman spectra were normalized to the intensity of C=O stretching vibration of ester groups at 1772 cm^−1^ [32].

### 2.7. Thermogravimetric Analysis

TGA was performed with the Q500 equipment (TA Instruments, New Castle, DE, USA) of TA Instruments under nitrogen or air atmospheres at a heating rate of 10 °C/min. The degradation temperatures of the distinct materials were determined, together with the exact SBA-15 amounts incorporated into the composites prepared by extrusion. These amounts were estimated as an average of values obtained from the two environments.

### 2.8. Differential Scanning Calorimetry

Calorimetric analyses were carried out in a TA Instruments Q100 calorimeter (TA Instruments, New Castle (DE) USA) connected to a cooling system and calibrated with different standards. The sample weights were around 3 mg. A temperature interval from −30 to 180 °C was studied at a heating rate of 10 °C/min. For the determination of the crystallinity, a value of 93.1 J/g was used as the enthalpy of fusion of a perfectly crystalline material [16,33].

### 2.9. Microhardness

A Vickers indenter attached to a Leitz microhardness tester (Leitz GmbH, Oberkochen, Germany) was used to perform micro-indentation measurements undertaken at 23 °C. A contact load of 0.98 N and a time of 25 s were employed. The microhardness (MH) value (in MPa) was calculated according to the following relationship [34,35]:

(1) $$\mathrm{MH}=2\sin68^{\circ }\left(\frac{P}{d^{2}}\right)$$

where *P* (in N) is the contact load and *d* (in mm) is the diagonal length of the projected indentation area. Diagonals were measured in the reflected light mode within 30 s of load removal using a digital eyepiece equipped with a Leitz computer-counter-printer (RZA-DO). This analysis provided preliminary information about dependence on aging time of mechanical features in the amorphous as-obtained materials.

## 3. Results and Discussion

### 3.1. Morphological and Structural Characteristics

**Figure 1:**
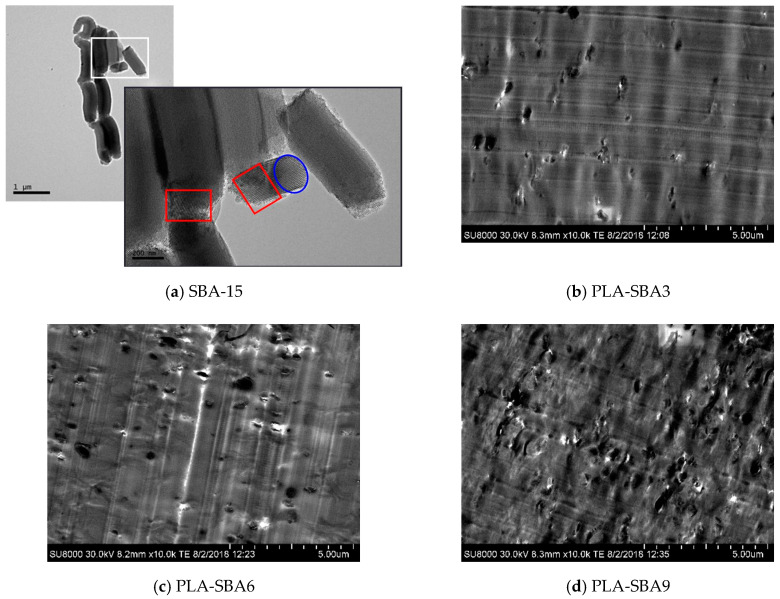
(**a**) TEM images of SBA-15 particles at a scale bar of 1 μm (left) and its magnification at 200 nm (right). FESEM micrographs for different composites: PLA-SBA3 (**b**), PLA-SBA6 (**c**), and PLA-SBA9 (**d**) at a scale bar of 5 μm.

Figure 1a shows TEM micrographs for the mesoporous SBA-15 silica used in this investigation. The left upper picture displays the distinctive vermicular elongated shape of these particles with an average width of 350 nm and length of 0.9 μm. The magnification shown in the right upper inset depicts the interior particle morphology consisting of the well-defined, uniform, and ordered channel structure (see within red squares) with hexagonal arrangements (see within the blue ellipse).

The micrographs depicted in Figure 1b–d correspond to the FESEM pictures for the PLA-SBA3, PLA-SBA6, and PLA-SBA9 composites. The suitable dispersion of SBA-15 particles within the PLA matrix and the non-existence of detectable bulky inorganic domains across the films at the different mesoporous silica contents are noticeable. Some agglomerates are, nevertheless, observed as SBA-15 composition was increased, but their sizes were not excessively large and they were found in a negligible proportion. These results seem to indicate a good contact at interfaces between silica particles and the PLA polymeric matrix despite their different chemical natures: hydrophobic for PLA and hydrophilic for the mesoporous silica.

**Figure 2:**
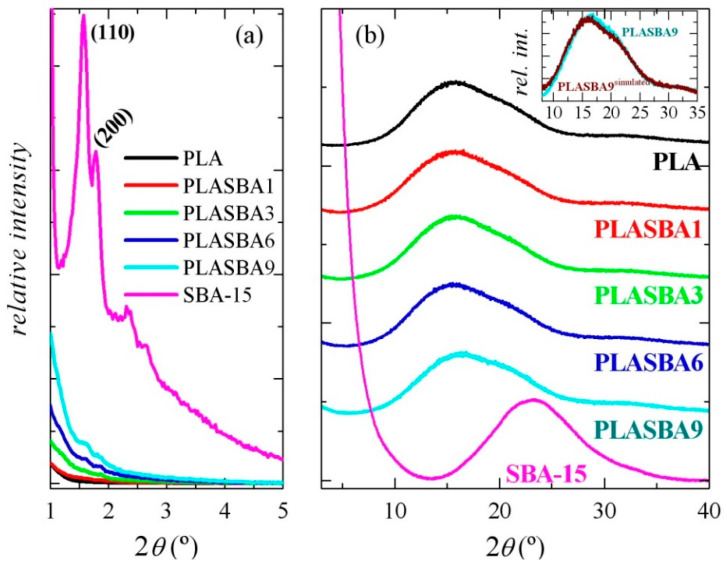
X-ray diffraction patterns at room temperature for pristine PLA, mesoporous SBA-15 particles, and the different composites based on PLA and distinct SBA-15 contents at middle (**a**) and wide angles (**b**). Inset: comparison between the experimental and simulated patterns (see text) for PLA-SBA9.

The ordered and well-defined channel structure with hexagonal arrangement presented by the mesoporous silica particles embedded in the PLA matrix was also deduced from the WAXD measurements at middle angles, as noticed in Figure 2a, for the different composites. The highly ordered hexagonal SBA-15 structure was characterized by three main diffraction peaks indexed as (100), (110), and (200) reflections, which are associated with its *p6mm* hexagonal symmetry [29]. It should be commented that the lower limit of the WAXD profiles represented in Figure 2a is 1° in the 2θ scale, so that the (100) reflections cannot be seen, and only the (110) and (200) main diffractions are the ones observed at this angular range. Furthermore, intensity of the specific SBA-15 reflections is significantly lowered in the composites, as much as the silica content incorporated decreases. Their position was not considerably changed although they appear to have been slightly altered. It has been described for hybrids based on polyethylene prepared by in situ polymerization [36,37,38,39] and on polypropylene ones, either obtained by in situ polymerization [40] or melt extrusion [41,42,43], that polymeric chains could grow up inside the hollow nanometric spaces that exist in both MCM-41 and SBA-15 particles during polymerization or could be pushed in those empty channels during the extrusion process. Accordingly, regularity at the mesoscale was not noticeably affected by the polymerization or shear forces applied during extrusion, and the hexagonal arrangement of the mesoporous silica channels was maintained.

Figure 2b shows that the processed PLA films quenched in the press were completely amorphous independently of the absence or incorporation of mesoporous SBA-15. This treatment was chosen for the films’ preparation to simulate the high cooling rates generally applied during common injection molding at the industrial scale. These composites of PLA with mesoporous silica have been rather unexplored [30] despite their great potential, and scarce knowledge has been actually established up to now.

The lower WAXD profile corresponded to the pristine SBA-15 silica, which also displayed its amorphous nature at short range. Thus, it was characterized by a broad diffraction centered at around 2θ = 23°. Accordingly, a shoulder overlapped to the main PLA amorphous halo became visible in the composites (Figure 2b) as the content in mesoporous particles increased.

The location of the PLA amorphous halo remained almost unchanged at 15.9° in the composites except for the PLA-SBA9, where it was shifted to 16.5°, i.e., to slightly higher 2θ values, as noticed in the inset of Figure 2b where the experimental pattern is compared with that simulated from the pristine constituents at their specific contents. This feature seems to indicate the establishment of some PLA–mesoporous silica interactions that led to a denser packing of the amorphous PLA chains and a slight modification of its intra and inter-chain distances in this composite. To get further information, Raman spectroscopy measurements were carried out.

**Figure 3:**
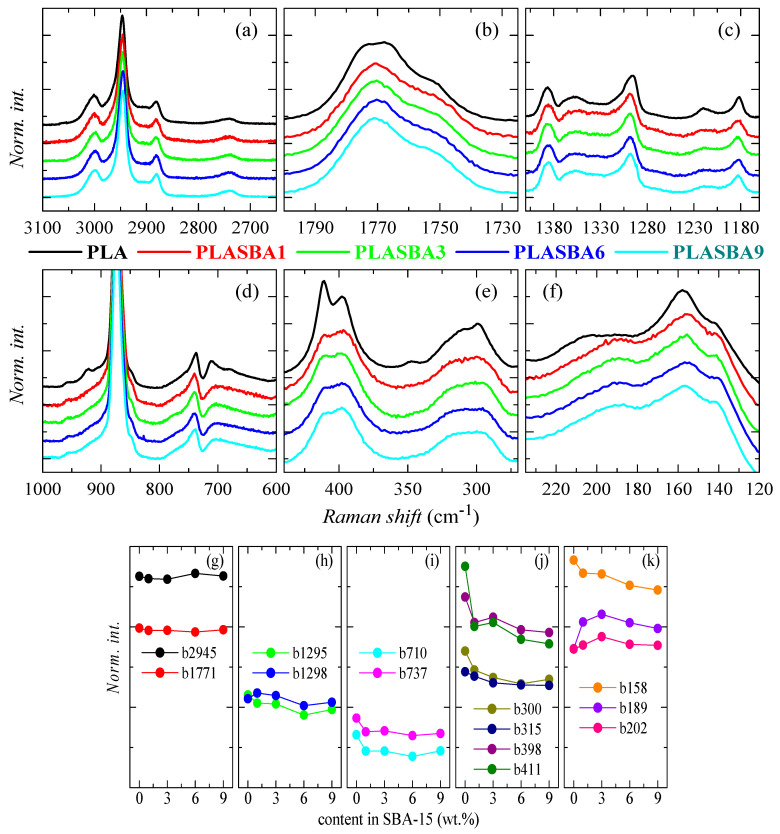
(**a**–**f**): Raman spectra for pristine PLA and its composites with mesoporous SBA-15 at different spectral regions; (**g**–**k**): variation with the SBA-15 content of the normalized intensity of the indicated bands.

Figure 3 shows the Raman shift for the different analyzed PLA materials at different vibrational zones.

Infrared and Raman spectroscopies have proven to be important tools for PLA characterization [12,16,44,45,46] to distinguish between different conformations and packing, together with distinct crystalline lattices and degrees of crystallinity. In an amorphous chain, the *tg’t* is the predominant conformational sequence for the three dihedral angles in a repeat unit of PLA [47,48].

Experimental investigations also clearly displayed that structural differences between the α and α’ phases were small but significant. The α phase consisted of 10_3_ helix conformations within an orthorhombic unit cell, as noted in the Introduction. In contrast, the α’ chain was a distorted helix and hexagonally packed. The main changes were, then, observed in vibrations involving carbonyl or methyl groups, which could be attributed to specific interactions concerning those functional groups. As depicted in Figure 2, all of the PLA samples under study were in an amorphous state. Consequently, no important variations were expected at first approximation.

The CH stretching region of the Raman spectra, 2800–3100 cm^−1^, is represented in Figure 3a. This zone remains rather inalterable by the incorporation of SBA-15 particles. Some differences have been found in the literature for specimens where the α phase was developed [18]. The carbonyl stretching region, 1700–1800 cm^−1^, has been described in PLA as precise for the assignment of the developed crystal polymorph [44,46]. Despite all the samples being amorphous, as seen in Figure 2b, a clear difference was observed between the neat PLA and the samples containing mesoporous SBA-15. This showed a broad band that consisted of three components, while the carbonyl band in the composites was formed by two noticeable contributions. This feature seems to indicate that the incorporation of silica changed the specific interactions between PLA chains. This might be associated with the establishment of some type of PLA–silica physical arrangement.

Figure 3c represents the spectral window from 1410 to 1260 cm^−1^. The band located at 1295 cm^−1^ for neat PLA was moved to 1298 cm^−1^ in the different composites. This change points out a denser packing in the chains of pristine PLA compared with that exhibited by the composites where SBA-15 particles were incorporated. A small reduction of its intensity was also observed in the composites at high silica contents. Similar features to these commented ones have been reported by comparison of Raman spectra for amorphous and semicrystalline PLA [49].

Particular sensitivity was also exhibited by shape and intensity of the 737 and 710 cm^−1^ bands, assignable to skeletal bending modes [18] (see Figure 3d). The relative intensity of the low frequency peak (710 cm^−1^) to the higher component was the greatest in the pristine PLA compared with the one shown by the composites. In addition, the band width of this low frequency component also decreased for the neat PLA. These characteristics seemed to again indicate that the amorphous chains in the pristine PLA were arranged in more compact conformations than PLA chains existing in composites with SBA-15 particles.

Another spectral region of particular interest was that where the deformation vibrations of the CCO and COC groups were coupled with CCH_3_ groups, typically seen at 420 and 290 cm^−1^ (see Figure 3e). The presence of the mesoporous silica significantly affected the shape of the vibrational bands appearing in this spectral window. An evident splitting was noticed at 411 and 398 cm^−1^, which involved the C=O in plane bending. This split has previously been observed in the spectrum of crystallized samples, and it has not been seen in that for amorphous samples [44]. This feature seems to demonstrate that the neat PLA exhibited conformations that boost a packing similar to that existing in α crystals. Additional variations were also shown at about 315 cm^−1^, indicating the existence of specific interactions between the carbonyl and methyl groups. This behavior was analogous to such exhibited between samples containing α or the α’ crystals [18]. Nevertheless, the PLA macrochains in all the analyzed samples were amorphous, as displayed Figure 2b.

Finally, a reduction in intensity and a displacement to lower frequencies were observed in the bands at about 200 and 150 cm^−1^, as deduced from Figure 3f. These facts were consistent with a less compact packing between the PLA chains in the composites because of the presence of the SBA-15 particles.

The previous aspects can also be clearly noticed in Figure 3g–k. Thus, the practical constancy of the band at 2945 cm^−1^ is observed in Figure 3g, as can that of the one at 1771 cm^−1^, whose area was considered for the normalization of the spectra. The shifting of the band located at 1295 cm^−1^ for neat PLA to 1298 cm^−1^ in the different composites can be deduced in Figure 3h from their initial decrease or increase, respectively. Figure 3i indicates the great sensitivity of the bands at 710 and 737 cm^−1^, the first being the one displaying the highest relative change. Figure 3j shows the variations of the bands at 411, 398, 315, and 300 cm^−1^, with an especially high decrease between PLA and the composites of the band at 411 cm^−1^. Finally, Figure 3k depicts the behavior of the bands at 202, 189, and 158 cm^−1^, characterized by an initial intensity increase for the first two bands and a very important decrease of the band at 158 cm^−1^ from neat PLA to the different composites.

In summary, the presence of SBA-15 particles seems to alter the packing within the PLA chains, probably due to the establishment of some kind of interactions between PLA and SBA-15 in the composites. The effects of these changes on the final properties and phase transitions of the PLA chains are examined shortly.

### 3.2. Thermal Stability

PLA undergoes hydrolytic degradation and mesoporous SBA-15 particles are easily hydrophilic. This PLA degradation is primarily important at high temperatures and leads to a reduction of its molecular weight and a loss of its overall properties. The influence of SBA-15 silica on PLA’s thermal stability is thus important to be examined because composites are prepared by extrusion at high temperature. Accordingly, this is analyzed next.

Mesoporous silicas have also been used, sometimes, as catalysts for decomposition. This effect was found by Marcilla et al. [50] when they used TGA to study the degradation of PE under N_2_ in the presence and absence of mesoporous MCM-41. Aguado et al. [51] showed the efficiency of mesoporous aluminosilicate MCM-41 as promoter for the degradation of polyolefins into liquid fuels. This role as a promoter of decomposition was also found when MCM-41 silica was used as a catalyst carrier and filler for in situ polymerized polyethylene-based composites employing either neat mesoporous particles or those decorated with undecenoic acid or silanes [37,52,53].

**Figure 4:**
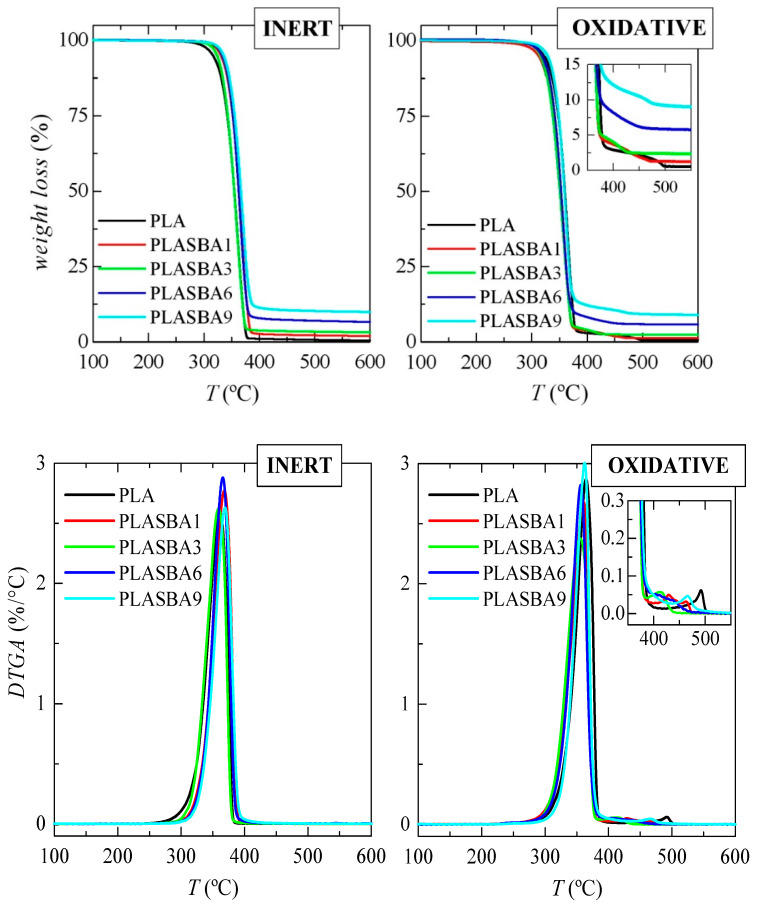
TGA curves under inert (**left**) and oxidative (**right**) atmospheres for the neat PLA and its composites with SBA-15 at different contents prepared by melt extrusion (top representations), as well as their corresponding derivative TGA (DTGA) curves (bottom representations).

Figure 4 shows the TGA curves and their derivatives (DTGA) under inert and oxidative conditions for these extruded materials. As observed, inert decomposition occurred in a single step from 300 to 400 °C. The presence of mesoporous silica did not greatly affect either how the process took place or its location. A shift to slightly higher temperature was, however, observed, as detailed in Table 1. Moreover, a narrowing of the degradation process in the composites can be deduced from the DTGA curves.

TGA curves under air exhibit two degradation processes. The main one took place at lower temperatures and involved the most of the weight loss, around 97%, and the secondary mechanism occurred just after the primary process ended. The incorporation of mesoporous silica did not have a significant role in the principal PLA decomposition stage, similarly to what was observed in the degradation performed under inert condition. Nevertheless, it seemed that presence of SBA-15 particles led to a little displacement to lower values of the temperature of maximum degradation and shortened the temperature interval at which the minor process occurred, as deduced from the DTGA curves under oxidative conditions.

All of these results demonstrated that presence of SBA-15 particles in a PLA matrix does not exert any significant catalytic effect in these composites. In fact, they become a little more stable if an inert atmosphere is used. It is interesting that a comparison of the TGA curves obtained under both environments shows that PLA degraded at an analogous range of temperature.

Furthermore, the exact amount of incorporated mesoporous silica was also estimated from these thermogravimetric curves achieved under the two environments. These data are also reported in Table 1—an average value was assessed. The fact that the two determinations were rather similar was an indirect effect of the homogeneity in the distribution of the content of the SBA-15 particles within the material at a given composition.

### 3.3. Existing Phases and Their Transitions

**Figure 5:**
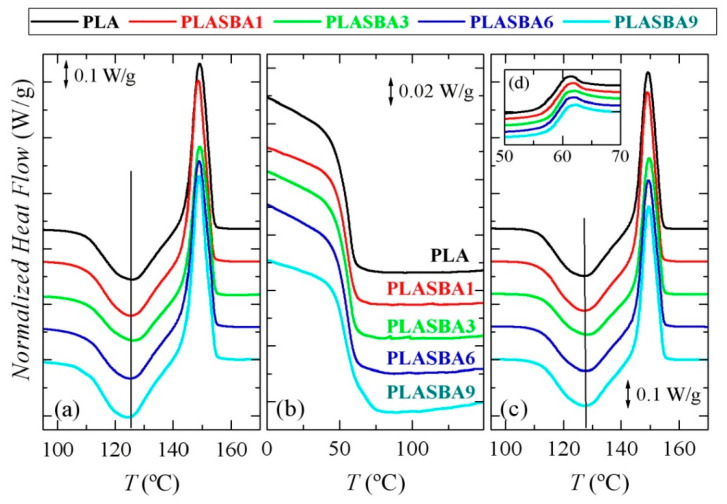
DSC curves performed at 10 °C/min for PLA and its composites with SBA-15, normalized to the actual PLA amount: (**a**) first heating, (**b**) cooling from the melt, (**c**) second heating, and (**d**) interval for glass transition during second heating.

Figure 5 shows the phase transitions observed in the neat PLA and its composites with SBA-15 during a first heating, cooling, and second heating runs performed at 10 °C/min. Knowledge of phase transition is mandatory to understand properties in polymers, but it is even more important in PLA because its glass transition undergoes physical aging and its crystallization takes place slowly, both facts exerting a key role in its dimensional stability. Analysis is now focused on the crystallization process since the main purpose of adding SBA-15 particles was to learn their influence on the ordering arrangement of PLA chains.

Figure 5a is related to the first heating experiments. Glass transition (not shown) appeared in all the samples at approximately 57 °C, i.e., the incorporation of mesoporous silica seemed not to greatly affect its location. At higher temperatures, the cold crystallization process of PLA was seen. It took place at 126.5 °C in all the samples with the exception of PLA-SBA9 where a shift to 124.5 °C occurred. At this highest content in mesoporous silica, a slight nucleating effect was thus observed when crystallization occurred from the glassy state. The intensity of cold crystallization remained rather constant independently of the SBA-15 content. Once this exothermic event finished, a melting process was initiated. It was characterized by the appearance of a unique peak whose maximum was seen at 149 °C in all the specimens. The equality of enthalpies involved in the cold crystallization and the subsequent melting process led to a zero neat total enthalpy of melting, a fact that, in agreement with the X-ray diffraction results, indicates the complete amorphous character of PLA in the neat polymer and as matrix in its composites after the quench applied in manufacturing of the films. It is remarkable that the PLA under analysis showed very close temperatures for the cold crystallization and the subsequent melting process. This feature was different to the exhibited by other PLAs reported in the literature [10,16] and implied some peculiarities concerning the type of polymorphs to be developed, as is commented upon below. Furthermore, the melting temperature, *T_m_*, of this PLA under study exhibited a value unusually low compared with other PLAs reported in the literature [10,16].

Figure 5b concerns the cooling process at 10 °C/min (note that the y-scale has been magnified by a factor of 5 in relation to the melting curves). The only clearly observed transition was the glass transition, which occurred at 55 °C. Its location did not change with the incorporation of the mesoporous SBA-15 particles, as already observed during the first melting. Nevertheless, the composite PLA-SBA9 (and also PLA-SBA6 in a very minor amount) additionally showed a rather small crystallization process that took place very close to the beginning of the glass transition. The slight nucleating effect observed during the first heating run for this SBA-15 content was again noticeable. Figure 5c displays the results found in the second heating process, now after a cooling rate of only 10 °C/min, i.e., considerably slower than that applied during initial film processing. The observed glass transition was now slightly moved to higher temperatures of approximately 59 °C, as seen in Figure 5d. The extension of physical aging was considerably reduced compared with that observed during the first heating since samples were just cooled. Concerning the cold crystallization of PLA, it should be noted that no important variations were seen after the cooling at 10 °C/min. The minimum appeared at 127 °C, and its location and intensity were not dependent on either the presence or content of mesoporous silica. After this crystallization, melting started, and its maximum appeared at 149 °C for all the specimens. The observed effect in the ordering of this PLA by an incorporation of 9 wt.% in mesoporous silica for the PLA-SBA9 composite encouraged a deeper analysis of the crystallization process. Thus, isothermal experiments were performed, either from the glass or molten states.

**Figure 6:**
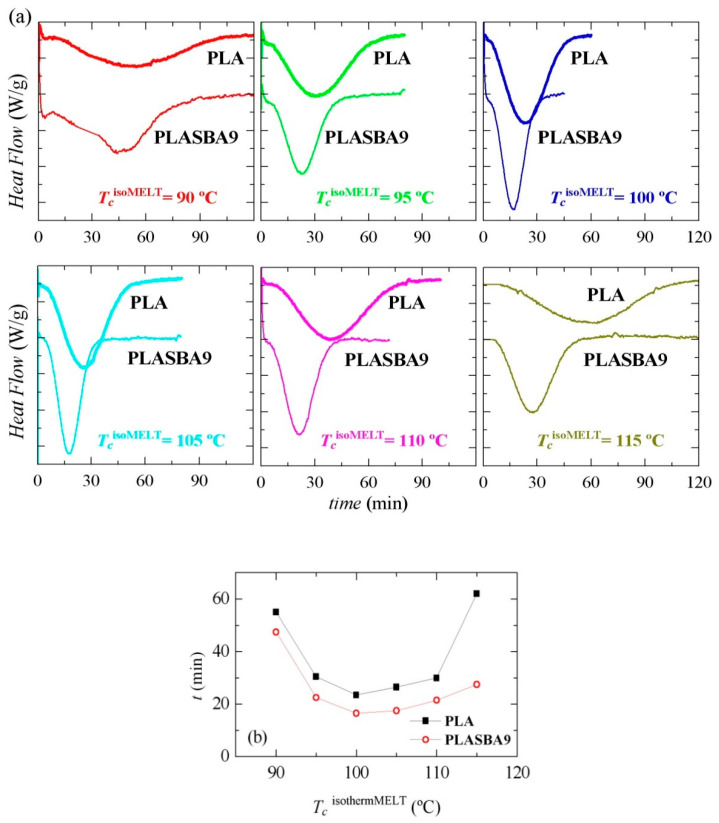
(**a**) Dependence of heat flow on time for isothermal crystallization from the melt, *T_c_*^isothermMELT^, at the indicated temperatures for PLA and PLA-SBA9. (**b**) Comparison of the peak crystallization times as a function *T_c_*^isothermMELT^ in PLA and PLA-SBA9.

Figure 6a shows a comparison between isothermal crystallization from the molten state for PLA and PLA-SBA9 at different temperatures.

It is noticeable that the presence of SBA-15 silica exerted an important effect and led to the acceleration of the PLA crystallization independently of the analyzed *T_c_*^isothermMELT^. Accordingly, the crystallization shifted to shorter times and was narrower in the PLA-SBA9 than in the pristine PLA at a given *T_c_*^isothermMELT^. The fastest crystallization temperature for the PLA chains from both specimens was 100 °C, as seen in Figure 6b. Anyway, the crystallization times involved, even at the maxima, were very long.

**Figure 7:**
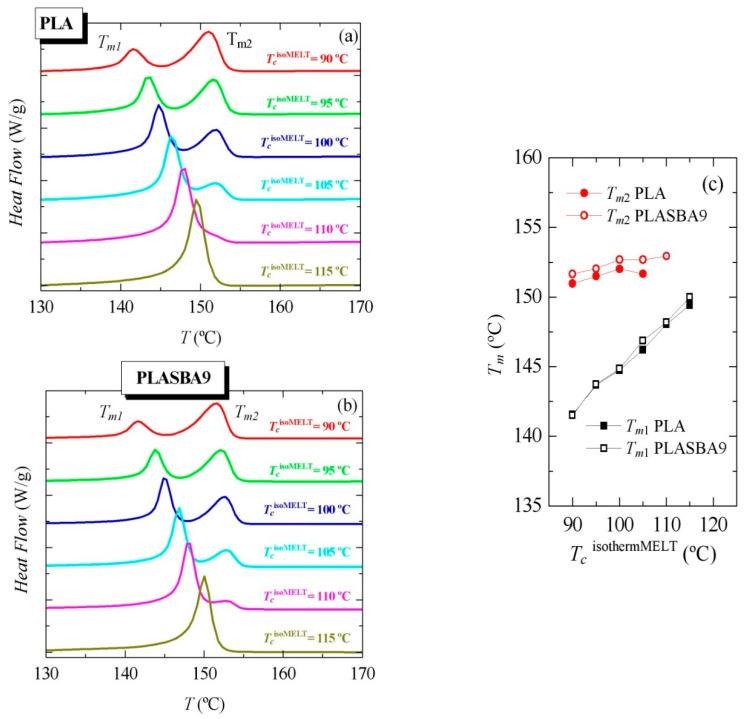
Effect of isothermal crystallization temperature from the melt, *T_c_*^isothermMELT^, on the melting behavior for (**a**) neat PLA and (**b**) its composite PLA-SBA9. (**c**) Relationship of melting temperatures with *T_c_*^isothermMELT^.

Figure 7 shows the successive melting processes after those isothermal crystallizations from the melt. Two different melting trends were seen with increasing *T_c_*^isothermMELT^ from 90 to 115 °C. At *T_c_*^isothermMELT^ < 110 °C, the DSC curves showed two well-defined endothermic peaks, *T_m_*_1_ and *T_m_*_2_ at low and high temperatures, respectively. The peak height of *T_m_*_2_ relative to *T_m_*_1_ decreased and shifted to lower temperatures with increasing *T_c_*^isothermMELT^, up to 110 °C in PLA and 115 °C in PLA-SBA9—temperatures at which *T_m_*_2_ merged with *T_m_*_1_. Therefore, at *T_c_*^isothermMELT^ = 115 °C, only a single *T_m_*_1_ melting peak was observed. The incorporation of mesoporous SBA-15 did not practically exert influence on the position of *T_m_*_1_, as seen in Figure 7c, its effect being more significant for *T_m_*_2_. The origin of the two melting temperatures is commented upon below.

**Figure 8:**
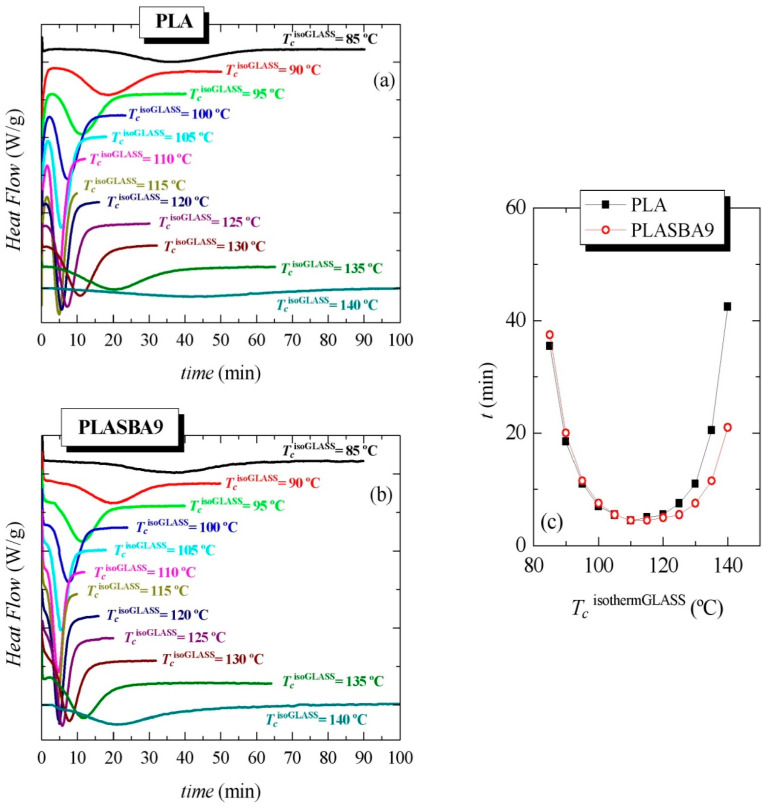
Dependence of heat flow on time for isothermal crystallization from the glass, *T_c_*^isothermGLASS^, at the indicated temperatures for (**a**) PLA and (**b**) PLA-SBA9. (**c**) Comparison of the peak crystallization times as a function of *T_c_*^isothermGLASS^ in PLA and PLA-SBA9.

Considering the rather long crystallization times for those isothermal experiments from the melt, additional isothermal crystallizations initiated from the amorphous glass were performed. Figure 8 shows the results found in a broad range of temperatures, named *T_c_*^isothermGLASS^, from 85 to 140 °C. As noticed in Figure 8a,b, independently of the incorporation of mesoporous SBA-15, crystallization was hindered at the lowest and the highest tested temperatures, requiring long times and exhibiting low extension.

Moreover, crystallization was moved to shorter times as temperature was increased, i.e., the ordering capability was sped up to reach a minimum (maximum rate) at around 110–115 °C, as seen in Figure 8c. Beyond those temperatures, the crystallization started to progressively slow down. This minimum was the result of the well-known opposite effects of the nucleation and transport terms in the crystallization rate.

Nevertheless, some differences were clearly noticed by effect of SBA-15’s presence. Thus, Figure 8c shows the time for peak crystallization as a function of *T_c_*^isothermGLASS^ in PLA and PLA-SBA9. It was observed that isothermal crystallization from the glassy state was slightly faster in pristine PLA than in PLA-SBA9 when it took place at temperatures lower than 100 °C, although variations became smaller as the temperature rose, being practically the same at temperatures of 105 and 110 °C. Above this isothermal temperature, the effect of SBA-15 became more important, the differences increasing as the isothermal crystallization temperature did, as can be seen in Figure 8c. In fact, the peak crystallization time was reduced to practically one half at 140 °C when comparing PLA and PLA-SBA9.

On the other hand, the inspection of Figure 6b and Figure 8c allows one to observe that PLA’s isothermal crystallization from the glass, i.e., along cold crystallization, was much faster than from the molten state. For instance, the times of peak crystallization were around four-to-five times smaller in the experiments from the glassy state than from the molten state. Those high differences were also responsible of the fact that the nucleating effect of mesoporous SBA-15 became more considerable when crystallization was initiated from the melt.

**Figure 9:**
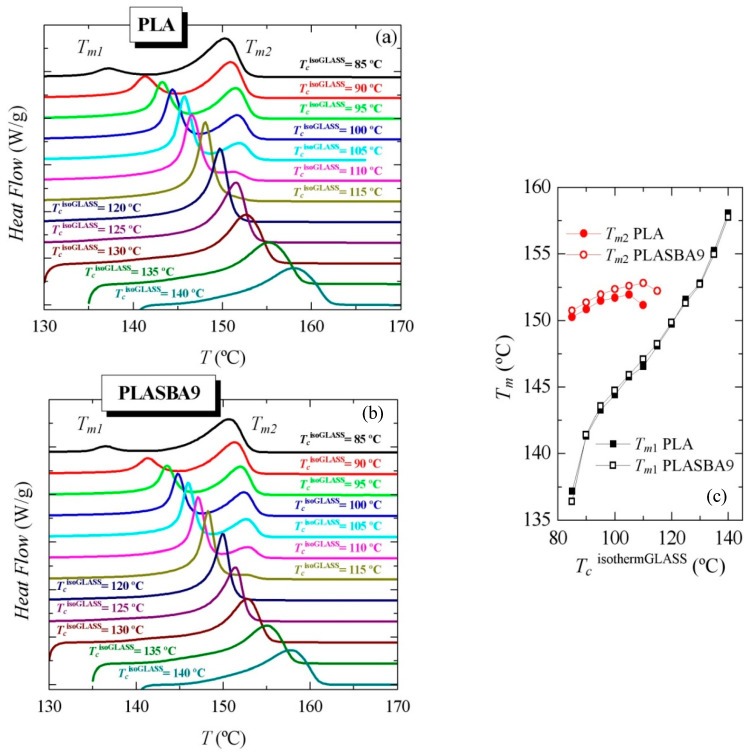
Effect of isothermal crystallization temperature from the glass, *T_c_*^isothermGLASS^, on the melting behavior for (**a**) neat PLA and (**b**) its composite PLA-SBA9. (**c**) Dependence of melting temperatures on *T_c_*^isothermGLASS^.

Figure 9 displays the characteristics found during the subsequent melting process after isothermal crystallization from the glass. Two different melting behaviors were again clearly noticeable with increasing *T_c_*^isothermGLASS^ from 85 to 140 °C. At *T_c_*^isothermGLASS^ < 120 °C, the DSC curves show two well-defined endothermic peaks: *T_m_*_1_ and *T_m_*_2_ at low and high temperatures, respectively. The peak height of *T_m_*_2_ relative to *T_m_*_1_ decreased and *T_m_*_2_ shifted to lower temperatures with increasing *T_c_*^isothermGLASS^ up to 120 °C, the temperature at which it merged with *T_m_*_1_. At *T_c_*^isothermGLASS^ > 120 °C, only a single *T_m_*_1_ melting peak appeared. The influence of presence of SBA-15 particles did not practically affect the position of *T_m_*_2_, as deduced from Figure 9c, but the dependence of *T_m_*_1_ on *T_c_* was rather pronounced.

Coming now to the origin of the two melting peaks, and as noted in the Introduction, two distinct crystalline polymorphs, labeled as α’ and α, could be developed under most processing conditions. Different ratios of both lattices can be achieved for PLA depending on the crystallization temperature, as reported in the literature [10,11,19]. These reports have indicated that when the crystallization occurs below 100 °C for the studied PLA sample, almost only the α’ crystalline lattice is formed, and when it takes place above 120 °C, the α form is exclusively developed. The coexistence of both types of crystallites appears in the interval between 100 and 120 °C, their ratio being mainly varied by crystallization temperature and intrinsic PLA characteristics. Was this behavior also noticeable for the present PLA sample?

It seemed that something different took place in this PLA and its composites with SBA-15 because the usual melting of the α’ crystals involved its recrystallization into the α modification, which is characterized by an exothermic event [16]. These characteristics were not observed in the results represented in Figures 7a,b and 9a,b [19]. It seems, therefore, that the found behavior was only related to the melting of the α crystalline polymorph [10]. The lower-temperature peak *T_m_*_1_ refers then to the melting of the original crystals developed during isothermal crystallization, while the higher-temperature endotherm *T_m_*_2_ refers to the melting of the recrystallized entities after thickening upon melting, with very little variation with *T_c_*. Finally, when the *T_c_*^isothermGLASS^ was above 120 °C, the pristine crystals were thick enough and only a melting component was observed.

**Figure 10:**
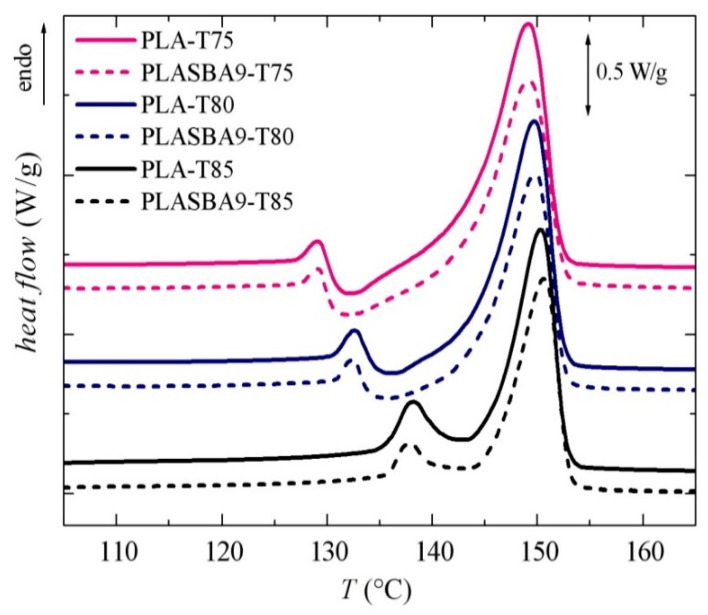
DSC melting curves after crystallization for 8 h from the glassy state at the indicated temperatures for the pristine PLA and its composite PLA-SBA9.

Interestingly, dependence of *T_m_*_1_ on *T_c_* seen in Figure 9c, shows a clear change of the slope below around 90 °C. Therefore, although the observation of the crystallization isotherm when crystallizing at temperatures below 85 °C was not possible due to the deterioration of the signal-to-noise ratio due to the relatively high crystallization times, additional experiments were performed by annealing for long times without registering the crystallization isotherm. Thus, Figure 10 shows the DSC melting curves after crystallization for 8 h at the indicated temperatures from the glassy state.

Now, the behavior at the crystallization temperatures of 75 and 80 °C was that commonly observed for the melting of the α’ crystals with its recrystallization into the α modification, characterized by an exothermic event. At 85 °C, that exothermic event was not exhibited, pointing out the important hindrance of α’ crystal formation at that relatively low crystallization temperature. If a certain amount of α’ crystallites had been developed, it was small, coexisting with α crystals, which existed as a major amount. The melting curve of this long crystallization at 85 °C was rather similar to that depicted in Figure 9a, where the total crystallization time was only 90 min.

Considering these results and the commented change of the slope in Figure 9c below around 90 °C, it can be tentatively said that α’ crystals were obtained for this PLA and its composite PLA-SBA9 when crystallizing below 80 °C, and the α modification was the only one obtained above 90 °C, probably with a mixture of the two forms in the approximate interval of crystallization temperatures from 80 to 90 °C.

**Figure 11:**
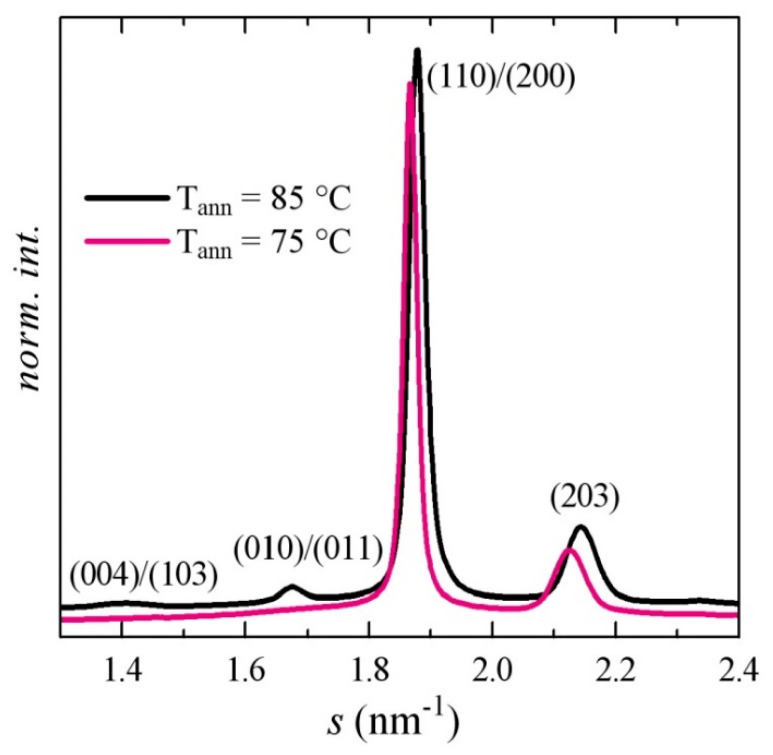
Synchrotron X-ray diffraction patterns at room temperature for PLA after crystallization for 8 h from the glassy state at the indicated temperatures. The Miller indices are indicated.

In order to ascertain that conclusion, X-ray diffraction experiments with synchrotron radiation were performed for samples crystallized at different temperatures and times. A comprehensive study is under progress for the analysis of the melting curves subsequent to those crystallizations. For the present purpose, the results shown in Figure 11 are revealing enough, displaying the diffractograms at room temperature for samples of the neat PLA crystallized at two temperatures: 75 and 85 °C. According to the literature [11,14,54], these profiles are typical for a majority of α’ crystallites in samples crystallized at 75 °C and of α crystals in samples crystallized at 85 °C. Here, they were characterized by the practical absence of reflections (103) and (010) for the α’ crystallites and a displacement to higher *s* values (lower spacings) for the (110)/(200) and (203) peaks for the α modification.

### 3.4. Mechanical Response

**Figure 12:**
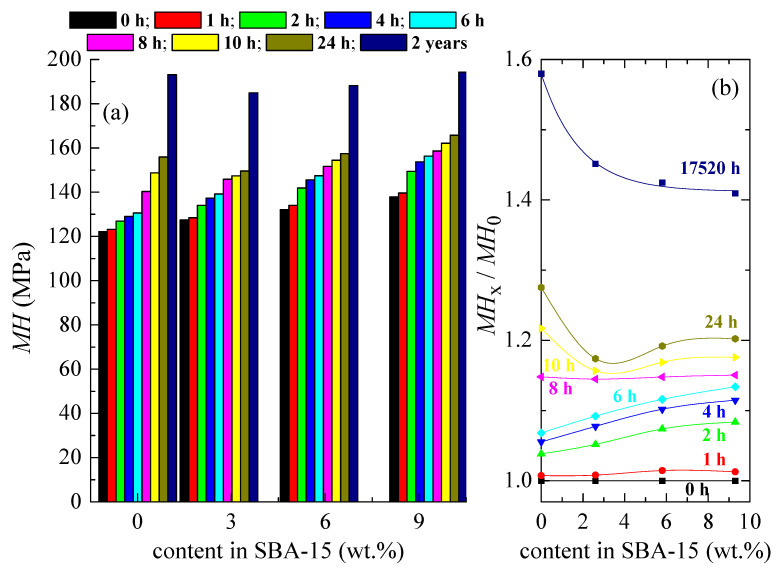
(**a**) Microhardness (MH) dependence on SBA-15 content at different aging times for neat PLA and its composites with silica processed from the melt. (**b**) Variation with silica content of the ratio between the actual MH and the initial value at each sample for the several analyzed aging times.

Microhardness measurements were performed to preliminarily estimate the mechanical response exhibited by these materials after their processing. As previously mentioned, all of them were amorphous. As seen in Figure 5d, an endothermic peak overlapped with the PLA glass transition related to its physical aging, even in those just-cooled specimens. This phenomenon, referred to as the molecular rearrangements of the amorphous PLA chains, takes place when its structure and, consequently, its thermodynamic variables (volume, enthalpy, and entropy) evolve toward the equilibrium [55,56]. These changes provoke variations with the aging time in the whole spectrum of properties until equilibrium is reached. Four weeks at room temperature is considered enough time [16] for the amorphous PLA phase to achieve equilibrium. This process could be accelerated if higher temperatures closer to but below T_g_ [55] are used. Figure 12 shows the MH values for PLA and some of its composites with SBA-15 at different aging times, as well as the variation with silica content of ratio between the actual MH and the initial value at each sample for the several analyzed aging times.

The just-processed specimens, as observed in Figure 12a, displayed a linear increase of MH with SBA-15 weight content, from 122 MPa for PLA to 138 MPa for PLA-SBA9. This fact points out the reinforcement role that mesoporous silica particles had in the amorphous glassy PLA macrochains. This tendency was expected since SBA-15 particles are harder than polymeric chains.

An analogous linear behavior (although MH values were raised for each sample with aging) was observed with aging time up to 8 h elapsed from processing. The reason why MH values for a specific sample were not kept constant with time was because physical aging took place in the polymeric chains (either in the pristine PLA or in the PLA matrix for the distinct composites). Tests were performed at a temperature below T_g_, and its amorphous glassy macromolecules were out of equilibrium and evolved to reach that state. PLA volume was then diminished, as previously mentioned, and its chains became denser and, consequently, more rigid during this structural recovery to equilibrium. Then, all mechanical parameters related to stiffness increased with aging time.

Nevertheless, the linear trend was not further accomplished at aging times higher than 8 h. In fact, values of the pristine PLA were larger than those in the PLA-SBA3 composites at 10 h, 24 h, and 2 years. The same feature was found in the MH of the PLA-SBA6 composite for an aging time of two years. Only the PLA-SBA9 material exhibited MH values higher than neat PLA in the whole interval of analyzed aging times. What happened?

Two aspects contributed to increase rigidity: the filler effect of SBA-15 particles and the physical aging in PLA chains. The former was expected to be constant with temperature, but the latter depended on the absence or presence of SBA-15 in the materials under analysis. That means, as deduced from Figure 12b, that PLA aged faster in the neat matrix than in the composites, and this rate was inversely proportional to the SBA-15 silica content. Accordingly, the contribution of the PLA physical aging at 10 h from processing or longer times to rigidity in the pristine matrix was able to overcome the filler effect of an SBA-15 incorporation of 3 wt.%. The same occurred in the PLA-SBA6 but now at longer aging times since a higher SBA-15 content was now incorporated. Rather analogous values were observed when PLA and PLA-SBA9 were compared after two years at room temperature from its processing, once physical aging process was supposedly finished. Accordingly, physical aging’s contribution to rigidity characteristics was really important in the pristine PLA, though it apparently secondary in the composites. However, this behavior was only valid for completely amorphous samples. MH dependence at room temperature will be different for this neat PLA and its composites if PLA crystallizes, either from the melt of from the glassy state, since other variables (crystallinity, crystallite size, type of polymorph developed, and the ratio between distinct crystalline lattices) will play a key role. Experiments are under progress for semicrystalline PLA and its materials with SBA-15 particles, all manufactured through extrusion.

## 4. Conclusions

Several composites based on an *L*-rich PLA with different contents of mesoporous SBA-15 were prepared by melt extrusion in order to evaluate the effect of mesoporous silica on the resultant PLA materials. These were completely amorphous since their processing as films consisted of a quenching from the melt to simulate the high cooling rates generally applied at the industrial scale. Their morphological aspects, changes in PLA phases, and their transitions, as well as some final properties, such as thermal stability and preliminary mechanical behavior, were thoroughly examined.

Morphological observations suggested a good contact at interfaces between the PLA matrix and the mesoporous silica particles. This was in agreement with the changes observed in the PLA packing, deduced from Raman experiments, associated with the establishment of some kind of interactions between PLA and SBA-15 in the composites.

The incorporation of SBA-15 particles into the PLA matrix did not exert any significant influence on the thermal decomposition of these composites under inert or oxidative atmospheres.

An important nucleation effect of the silica in the PLA was, however, found in non-isothermal experiments and also under isothermal crystallization, either from the melt or from the glassy state. Isothermal crystallization from the glass was considerably faster than from the molten state, with peak crystallization times around four-to-five times smaller in the former. These high differences were also responsible for the fact that the nucleating effect of mesoporous SBA-15 became more considerable when crystallization was initiated from the melt.

Cold PLA crystallization and its subsequent melting took places very close one another for the PLA under analysis, this feature being different to that exhibited by other PLAs reported in the literature. This behavior implied some peculiarities concerning the type of polymorphs to be developed. Thus, all the isothermal experiments that allowed for the observation of the crystallization isotherm with a sufficient signal-to-noise ratio led to the exclusive formation of the α modification. These isothermal experiments showed a minimum peak crystallization time (maximum rate) at around 110 °C, this minimum being the result of the well-known opposite effects of the nucleation and transport terms in the crystallization rate.

The development of the α’ crystals required a crystallization at temperatures below 80 °C for long times. Thus, α’ crystals were obtained for this PLA and its composites with SBA-15 particles when the PLA macrochains were isothermally maintained below 80 °C for a long enough time (eight hours), while the α modification was the only polymorph obtained above 90 °C, probably with a mixture of the two forms in the approximate interval of crystallization temperatures from 80 to 85 °C. These remarks were ascertained based on X-ray diffraction of samples from neat PLA. Its profiles demonstrated a majority of α’ modification for the PLA crystallized at 75 °C and of α crystals for that crystallized 85 °C.

Microhardness measurements of these amorphous PLA–SBA-15 composites interestingly indicated that rigidity behavior based on silica composition depended on two factors: the reinforcement effect of mesoporous particles on PLA and the extent of physical aging within a polymeric matrix. At short aging times after processing, the former had a prevailing role, and a linear increase with SBA-15 content was clearly noticeable. Nevertheless, the PLA physical aging of the neat matrix overcame the SBA-15’s effect as a filler for the composites with the lowest silica contents at aging times longer than 10 h. Accordingly, the influence of physical aging was really important in the pristine PLA and apparently played a secondary role in the composites.

## Figures and Tables

**Table 1:** TGA results under nitrogen and air atmospheres for neat PLA and composites prepared by melt extrusion: temperature of a loss weight of 10% (T_10%_) and temperature at the maximum (T_max_), together with the SBA-15 wt.% content in both environments and its average.

Sample	Average SBA-15 wt.% Content	Inert Atmosphere			Oxidative Atmosphere		
		T_10%_ (°C)	T_max_ (°C)	SBA-15 wt.% Content	T_10%_ (°C)	T_max_ (°C)	SBA-15 wt.% Content
PLA	0	328.0	361.2	0	333.0	364.6	0
PLA-SBA1	1.4	339.5	368.0	1.6	324.0	360.6	1.3
PLA-SBA3	2.6	331.0	359.0	2.8	325.0	355.4	2.4
PLA-SBA6	5.8	341.0	365.6	6.1	328.5	356.2	5.6
PLA-SBA9	9.3	343.0	369.4	9.6	336.5	362.4	8.9
